# OptiDose: Computing the Individualized Optimal Drug Dosing Regimen Using Optimal Control

**DOI:** 10.1007/s10957-021-01819-w

**Published:** 2021-02-24

**Authors:** Freya Bachmann, Gilbert Koch, Marc Pfister, Gabor Szinnai, Johannes Schropp

**Affiliations:** 1 Department of Mathematics and Statistics, University of Konstanz, Konstanz, Germany; 2 Pediatric Pharmacology and Pharmacometrics, University Children’s Hospital Basel, University of Basel, Basel, Switzerland; 3 Pediatric Endocrinology and Diabetology, University Children’s Hospital Basel, University of Basel, Basel, Switzerland

**Keywords:** Optimal control, Model predictive control, Quasi-Newton methods, Individualized optimal drug dosing, Pharmacokinetic–pharmacodynamic models, 49K15, 65K10, 92C45, 92C50

## Abstract

Providing the optimal dosing strategy of a drug for an individual patient is an important task in pharmaceutical sciences and daily clinical application. We developed and validated an optimal dosing algorithm (OptiDose) that computes the optimal individualized dosing regimen for pharmacokinetic–pharmacodynamic models in substantially different scenarios with various routes of administration by solving an optimal control problem. The aim is to compute a control that brings the underlying system as closely as possible to a desired reference function by minimizing a cost functional. In pharmacokinetic–pharmacodynamic modeling, the controls are the administered doses and the reference function can be the disease progression. Drug administration at certain time points provides a finite number of discrete controls, the drug doses, determining the drug concentration and its effect on the disease progression. Consequently, rewriting the cost functional gives a finite-dimensional optimal control problem depending only on the doses. Adjoint techniques allow to compute the gradient of the cost functional efficiently. This admits to solve the optimal control problem with robust algorithms such as quasi-Newton methods from finite-dimensional optimization. OptiDose is applied to three relevant but substantially different pharmacokinetic–pharmacodynamic examples.

## Introduction

An optimal drug dosing regimen is a prerequisite to provide the best possible care for every individual patient. However, a diversity of individual factors need to be considered including current disease state, patient characteristics and the clinical goal for this patient. In pediatric patients, developmental changes have to be incorporated additionally. In (preterm) neonates, we face even more difficulties because fast maturation processes start immediately after birth which impact the drug effect, see, e.g., [1–3]. Therefore, it is essential to support clinicians with sophisticated mathematical methods to compute the optimal dosing regimen for every individual patient.

Mathematical modeling has become an essential tool in drug developing industries and clinical pharmacology departments in hospitals. The US Food and Drug Administration recognized such computational modeling and simulation tools as an improvement in the efficiency for developing safe and effective drugs [4], especially the so-called pharmacokinetics (PK) and pharmacodynamics (PD) models [5–7], a combination of mathematical and statistical methods, incorporate biological, physiological and pharmacological behavior. Roughly speaking, the PK deals with the distribution and elimination of a drug and the PD characterizes the effect of the drug on a specific target. PKPD models are formulated with a system of nonlinear differential equations [7–10].

Up to now, the computation of a “good” dosing regimen for an individual patient is a laborious and biased task in clinical pharmacology. Often, a large number of simulations for varying dosing regimens are performed with the developed PKPD model and the “best” dosing regimen is selected “by hand.” In contrast, e.g., in the development process of oncology drugs [11], optimal control approaches are already used to increase the probability of successful phase 2 clinical trials [12]. However, the research questions in such clinical trials during drug development may differ from the goals in clinical application, and therefore, often the drug concentration of potential drug candidates is optimized and not the doses itself that cause the drug concentration.

In clinics, the goal for the individual patient is usually clearly defined by the physician. For example, in hormonal diseases the aim is not always to return the hormone levels as quickly as possible to the normal range but to follow a moderate disease reduction over several weeks. Another situation is to achieve a specific concentration of a target (e.g., a drug–receptor complex) having the most beneficial impact on the disease [13].

Although control theory is widely applied in many different engineering fields such as aerospace (the field where it was originally developed by Pontryagin [14]) or economics, its application in daily clinical practice is still quite rare. Various reasons are possible, e.g., many drugs are promoted as “one size fits all,” and therefore, individual patient characteristics are completely ignored. Another reason might be much simpler; currently, to our knowledge no software for solving optimal control problems (OCP) is available that is custom-built for PKPD models and addresses the needs in daily clinical application.

In this paper, we develop a mathematical OCP that is especially designed for PKPD models and name the software OptiDose. In contrast with many applications [12, 15, 16], we are not using the time course of the drug as a continuous control. We directly optimize the doses for a given time schedule which is the clinical situation for patients treated with oral, subcutaneous or intravenous bolus administration. This allows to construct a finite-dimensional reduced OCP which can be solved using robust algorithms from finite-dimensional optimization such as quasi-Newton methods.

Moreover, we will apply nonlinear model predictive control (NMPC), in engineering also called closed-loop strategy, see [17] for theoretical details and [18] for an application to hemodialysis. This meets clinical needs as it allows to adapt patient parameters during the optimization if the covariates change over time, e.g., due to maturation processes or sudden changes in the disease characteristics, or if more clinical measurements are available. In addition, this strategy enables to react to unforeseen events such as missed or wrong doses.

Finally, we present three examples of different complexity, a biomarker indirect response model, a tumor growth inhibition model and a model characterizing various binding dynamics of a bispecific monoclonal antibody from immuno-oncology.

## The Pharmacokinetic–Pharmacodynamic Model

In this section, we present the pharmacokinetic–pharmacodynamic (PKPD) model, usually a system of nonlinear ordinary differential equations describing the dynamics of a certain disease and the action of a drug, and discuss its unique solvability.

Let us consider the time interval [0, *T*] with (possibly large) final time $T > 0$. We assume that for $i = 1,\dotsc ,m$ with $m \in {\mathbb {N}}$ the drug doses $u_i$ are administered $n_i \in {\mathbb {N}}$ times at specific time points $t_{i,l}$, $l=1, \ldots , n_i$ satisfying

$$\begin{aligned} 0 \le t_{1,1}< \ldots< t_{1,n_1}< \ldots< t_{m,1}< \ldots< t_{m,n_m} < T \end{aligned}$$

orally or subcutaneously (SC), as intravenous (IV) bolus or as IV infusion with a constant infusion rate for a certain duration $\varDelta t_{i}$, $i = 1,\ldots ,m$. In accordance with the typical notation in optimal control, we introduce $u=(u_1 , \ldots , u_m)$ and the associated finite-dimensional control space $U= {\mathbb {R}}^m$. As only nonnegative doses or rates with a given upper bound $u_{\mathrm{max}}\in {\mathbb {R}}^m, u_{\mathrm{max}}> 0$ can be administered, we define the convex and compact admissible subset

$$\begin{aligned} U_{{\mathrm{ad}}}= \{ {u}\in U\, | \, 0 \le {u}\le u_{\mathrm{max}}\} \subset U\end{aligned}$$

where ’$\le$’ denotes the componentwise comparison. Let ${u}\in U_{{\mathrm{ad}}}$ be arbitrarily chosen. Then, the PKPD model, in the context of optimal control also called the state equation, describes the model state

1 $$\begin{aligned} y'(t)&= g(t,\theta ,{u},y(t)), \qquad t \in \, ]0,T] \text{ almost } \text{ everywhere } \nonumber \\ y(0)&= {y_0}( \theta ) . \end{aligned}$$

Here $g :[0,T] \times \varTheta \times U\times {\mathbb {R}}^n \rightarrow {\mathbb {R}}^n$ is the PKPD mechanism, $\theta$ the model parameter in the set $\varTheta$ of admissible model parameters, ${y_0}(\theta )\in {\mathbb {R}}^n$ the initial value and $u=(u_1, \ldots , u_m ) \in U_{{\mathrm{ad}}}$ denotes the administered doses.

A reliable PKPD model (1) for a population with the same disease has to be validated thoroughly from measured data, and the model parameter $\theta$ has to be estimated reasonably before addressing the optimal dosing problem. In the optimal dosing step, the parameter $\theta$ is fixed, i.e., $\theta = \theta _{ind}$ for a certain individual in a population or $\theta = \theta _{av}$ characterizing the average value of a population. Without loss of generality, we thus use

2 $$\begin{aligned} y'(t)&= g(t,{u},y(t)), \qquad t \in \, ]0,T] \text{ almost } \text{ everywhere } \nonumber \\ y(0)&= {y_0}\end{aligned}$$

with $g :[0,T] \times U\times {\mathbb {R}}^n \rightarrow {\mathbb {R}}^n$ as PKPD model and initial value $y_0$. Moreover, PKPD models inherit an additive structure

3 $$\begin{aligned} g(t,{u},y) = \tilde{g}(t,y) + r(t,{u}) \end{aligned}$$

between state *y* and control *u* from their design principles. In (3), $\tilde{g} :[0,T] \times {\mathbb {R}}^n \rightarrow {\mathbb {R}}^n$ describes the pharmacological mechanism and $r :[0,T] \times U\rightarrow {\mathbb {R}}^n$ the dosing regimen. For the dosing, we encounter oral or SC administration into an absorption compartment, or IV bolus injection or IV infusion for a certain duration $\varDelta t_i$ into the central compartment characterizing the blood in the body. This leads to

4 $$\begin{aligned} r(t,{u})= & {} In (t,u) \, e^{j_0} \nonumber \\= & {} {\left\{ \begin{array}{ll} \sum _{i=1}^m u_i \sum _{l=1}^{n_i} \delta (t-t_{i,l}) \, e^{j_0} \qquad \qquad \; \;\text { for oral / SC administration} \\ \sum _{i=1}^m \frac{u_i}{V} \sum _{l=1}^{n_i} \delta (t-t_{i,l}) \, e^{j_0} \qquad \qquad \; \, \text { for IV bolus} \\ \sum _{i=1}^m \frac{u_i}{V \varDelta t_i} \sum _{l=1}^{n_i} {\mathbb {1}}_{[t_{i,l},t_{i,l}+\varDelta t_i]} (t) \, e^{j_0} \quad \text{ for } \text{ IV } \text{ infusion } \\ \end{array}\right. } \end{aligned}$$

where *In*(*t*, *u*) denotes the input function depending on the dosing regimen *u*, $\delta$ is the Dirac distribution, $j_0$ is the component of *y* to which the dose will be added to and $e^{j_0}$ denotes the $j_0$-th unit vector. The parameter *V* describes the volume of distribution of the body for the specific drug.

Equation (4) implies that solutions of (2) have jumps in the $j_0$-th component with jump conditions

5 $$\begin{aligned} y_{j_0}(t_{i,l}^+) - y_{j_0}(t_{i,l}^-) = {\left\{ \begin{array}{ll} u_i \qquad \; \, \text { for oral } / \text {SC administration} \\ \frac{u_i}{V} \qquad \; \text { for IV bolus} \\ \end{array}\right. } \end{aligned}$$

for $l= 1, \ldots ,n_i$, $i = 1,\ldots ,m$ where $t_{i,l}^+$ and $t_{i,l}^-$ denote the limits at the dosing time point $t_{i,l}$ from above and below, respectively. As a result, these routes of administration lead to impulse ordinary differential equations (ODEs), see [19]. In PKPD modeling, these impulse ODEs are handled by stopping the integration process at every dosing time point, adding the dose to the corresponding state (e.g., an absorption or central compartment) and continuing with the integration to the next dosing time point. In contrast with that, the IV infusion administration yields an OCP with continuous piecewise constant controls where no impulse control is present.

### Unique Solution of the State Equation

A minimal assumption in PKPD analysis is that for every dosing regimen there is exactly one solution of the state equation which, in addition, has to be sufficiently smooth. To do so, we formally modify our PKPD model (2) by replacing the Dirac distribution $\delta (t-t_{i,l} )$ in *r* by the scaled indicator function $\frac{1}{\epsilon } {\mathbb {1}}_{[t_{i,l} , t_{i,l} +\epsilon ]} (t)$. Hence, instead of oral / SC and IV bolus administration we apply a short infusion of length $\epsilon$. This leads to

6 $$\begin{aligned} g(t,{u},y) = \tilde{g}(t,y) + \tilde{r}(t,{u}) \end{aligned}$$

with

7 $$\begin{aligned} \tilde{r}(t,{u})= & {} \tilde{In} (t,u) \, e^{j_0} \nonumber \\= & {} {\left\{ \begin{array}{ll} \sum _{i=1}^m \frac{u_i}{\epsilon } \sum _{l=1}^{n_i} {\mathbb {1}}_{[t_{i,l}, t_{i,l} +\epsilon ]} (t) \, e^{j_0} \qquad \quad \, \text{ for } \text{ oral } \text{/ } \text{ SC } \text{ administration } \\ \sum _{i=1}^m \frac{u_i}{V \epsilon } \sum _{l=1}^{n_i} {\mathbb {1}}_{[t_{i,l}, t_{i,l} +\epsilon ]} (t) \, e^{j_0} \qquad \; \quad \text{ for } \text{ IV } \text{ bolus } \\ \sum _{i=1}^m \frac{u_i}{V \varDelta t_i} \sum _{l=1}^{n_i} {\mathbb {1}}_{[t_{i,l},t_{i,l}+\varDelta t_i]} (t) \, e^{j_0} \quad \text{ for } \text{ IV } \text{ infusion } \\ \end{array}\right. } \end{aligned}$$

For any ${u}\in U_{{\mathrm{ad}}}$ the function $\tilde{r}(\cdot ,u)$ is a step function and $\tilde{r}(t, \cdot )$ is linear in *u* for $t \in [0,T]$. We assume the pharmacological mechanism $\tilde{g}$ to be continuously differentiable and globally Lipschitz continuous with respect to *y*. Then iterative application of the Picard–Lindelöf theorem implies that for any *u* there is a unique solution $y \in C([0,T], {\mathbb {R}}^n )$ which is continuously differentiable when restricted to any time interval $I \subset [0,T ]$ on which $\tilde{r}(t,u)$, $t \in I$ is constant. Due to Example 6.2.5 in [20], the weak derivative of *y* exists and $y \in Y :=H^1(0,T; {\mathbb {R}}^n ) \cap C([0,T], {\mathbb {R}}^n )$ follows for the modified state equation (2), (6), (7).

Although mathematically different, from a clinical perspective the two dosing formulas (4) and (7) are practically equivalent.

## The Optimal Control Problem

Our goal is to achieve a certain outcome of the disease, e.g., a normal level of a hormone or tumor eradication. This desired disease progression is specified by a reference function . Then, we characterize optimality by minimizing the cost functional $J :Y \times U \rightarrow {\mathbb {R}},$8with $\alpha =( \alpha _1 , \ldots , \alpha _m) \ge 0$ and a $C^1$-functional $h :{\mathbb {R}}^n \rightarrow {\mathbb {R}}$ describing the actual state of the patient resulting from a particular dosing regimen ${u}$. In oncology, *h* could be the sum of proliferating and different stages of apoptotic tumor cells. The standard recommendation is $\alpha = 0$, but for some PKPD models it can be useful to add a small $\alpha > 0$ in favor of a linear dependency [12] to lower drug doses.

To formulate the optimal control problem, we follow the approach of [21], since it provides direct access to the adjoint and an efficient method to compute the gradient of the cost functional. The regularized PKPD model (2), (6), (7) is rewritten as equality constraint, i.e.,

$$\begin{aligned} e :Y \times U \rightarrow Z, \quad e(y,{u}) = \begin{pmatrix} e_1(y,{u}) \\ e_2(y,{u}) \end{pmatrix} =\begin{pmatrix} y' - g(\cdot ,{u},y) \\ y(0) - {y_0}\end{pmatrix} = 0 \in Z \end{aligned}$$

for $Z := L^2(0,T;{\mathbb {R}}^n) \times {\mathbb {R}}^n$. Then, the OCP reads 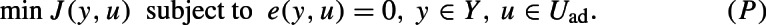


In the sequel, we work with the following set of assumptions:

**Assumption Optimal Control Problem**

1. $U_{{\mathrm{ad}}}\subset U$ is convex, bounded and closed.
2. $J :Y \times U \rightarrow {\mathbb {R}}$, $e :Y \times U \rightarrow Z$ are continuously Fréchet differentiable and *U*, *Y*, *Z* are Banach spaces.
3. For all ${u}\in \tilde{U}$ in a neighborhood $\tilde{U} \subset U$ of $U_{{\mathrm{ad}}}$, the state equation $e(y,{u}) = 0$ has a unique solution $y = y({u}) \in Y$.
4. $\frac{\partial }{\partial y} e(y({u}),{u}) \in {\mathcal {L}}(Y,Z)$ has a bounded inverse for all ${u}\in \tilde{U} \supset U_{{\mathrm{ad}}}$.

The assumptions follow the framework introduced in [21, Ch. 1.7.2], and the additional property $U_{{\mathrm{ad}}}\subset U$ bounded originates from the design of OCPs in PKPD modeling. Assumption 1) holds by definition of $U_{{\mathrm{ad}}}$, 2) and 3) are fulfilled due to the smoothness assumptions of $\tilde{g}$, *h* and the properties of the step function $\tilde{r}$. Computing the Fréchet derivative in assumption 4) for arbitrary but fixed ${u}\in \tilde{U}$ yields the operator:

$$\begin{aligned}&T({u}) :Y \rightarrow Z, \\&T({u}) \, y_{\delta }= \left( \frac{\partial }{\partial y}e (y({u}),{u})\right) \, y_{\delta }= \begin{pmatrix} y_{\delta }' - \frac{\partial }{\partial y}g(\cdot , {u}, y({u})) \, y_{\delta }\\ y_{\delta }(0) \end{pmatrix} \in Z \end{aligned}$$

Obviously, $T({u})$ is a linear operator which is bijective due to the Picard–Lindelöf theorem and the approximation of $L^2$-functions by continuous functions. Moreover, the Lipschitz continuity of *g* yields that $\Vert {\frac{\partial }{\partial y}g(\cdot , {u}, y({u})) }\Vert$ is bounded, and therefore, $T({u})$ is continuous. Then assumption 4) follows by the bounded inverse theorem, cf. [22].

### Existence of Optimal Controls

#### Definition 3.1

A control $\bar{{u}} \in U_{{\mathrm{ad}}}$ is called optimal for (*P*) and $\bar{y} = y(\bar{{u}}) \in Y$ is called the associated optimal state if

$$\begin{aligned} J(\bar{y}, \bar{{u}}) \le J(y({u}),{u}) \; \text{ for } \text{ all } {u}\in U_{{\mathrm{ad}}}. \end{aligned}$$

The assumptions of the OCP admit to introduce the reduced cost functional

$$\begin{aligned} \hat{J}({u}) := J(y({u}),{u}) \end{aligned}$$

and the reduced optimal control problem 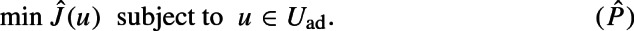


The existence of an optimal control $\bar{{u}}$ then follows from the compactness of $U_{{\mathrm{ad}}}\subset {\mathbb {R}}^m$ and the Weierstraß theorem, since the map $\hat{J} :U_{{\mathrm{ad}}}\rightarrow {\mathbb {R}}$ is continuously differentiable as the solution operator of the state equation ${u}\in \tilde{U} \mapsto y({u}) \in Y$ is continuously differentiable by the implicit function theorem and the assumptions of the OCP.

#### Remark 3.1

Due to the nonlinearity of the PKPD model and the lacking strictness of the convexity of *J*, we cannot guarantee uniqueness of the optimal control in general.

Theorem 5.2.2 in [23] ensures that if $\bar{{u}} \in U_{{\mathrm{ad}}}$ is a local solution of the reduced problem ($\hat{P}$), then $\bar{{u}}$ satisfies the variational inequality

9 $$\begin{aligned} \langle {\nabla \hat{J}(\bar{{u}}),{u}-\bar{{u}}}\rangle _{U} \ge 0 \quad \text{ for } \text{ all } {u}\in U_{{\mathrm{ad}}}. \end{aligned}$$

Now, we will derive necessary optimality conditions for a local solution $\bar{u} \in U_{{\mathrm{ad}}}$.

### Necessary First-Order Optimality Conditions

Following the notation in [21, Ch. 1.6.4], we define the Lagrange function associated with (*P*):

$$\begin{aligned} L :Y \times U \times Z \rightarrow {\mathbb {R}}, \quad L(y,{u},p) =J(y,{u}) + \langle {p,e(y,{u})}\rangle _{Z} \end{aligned}$$

Here, we identified *Z* with its dual space $Z^*$ and $p=(\tilde{p},\tilde{p}_0)$ is the so-called adjoint state. Moreover, as $\tilde{p}_0 = \tilde{p}(0)$ holds we write $p=:\tilde{p}$. The inner product $\langle { \cdot , \cdot }\rangle _Z$ is given by

$$\begin{aligned} \langle {\tilde{p},e(y,{u})}\rangle _{Z} = \langle {\tilde{p},e_1(y,{u})}\rangle _{L^2(0,T;{\mathbb {R}}^n)} +\langle {\tilde{p}(0),e_2(y,{u})}\rangle _{{\mathbb {R}}^n}. \end{aligned}$$

If $(\bar{y}, \bar{{u}})$ is an optimal solution to the problem (*P*), then there exists a Lagrange multiplier $\bar{p} \in Z$ such that the following optimality conditions hold, also called the KKT conditions after Karush, Kuhn and Tucker [21, Ch. 1.7.2]:

$$\begin{aligned} \forall {u}\in U_{{\mathrm{ad}}}: \left\langle \frac{\partial }{\partial u} L(\bar{y}, \bar{{u}}, \bar{p}), {u}- \bar{{u}}\right\rangle _U&\ge 0 \\ \frac{\partial }{\partial y}L(\bar{y}, \bar{{u}}, \bar{p})&= 0 \\ \frac{\partial }{\partial p}L(\bar{y}, \bar{{u}}, \bar{p})&= e(\bar{y}, \bar{{u}}) = 0 \end{aligned}$$

For arbitrary ${u}_\delta \in {\mathbb {R}}^m$, we compute the Fréchet derivative

10 $$\begin{aligned} \frac{\partial }{\partial u} L(y,{u},p) \, {u}_\delta&= \left\langle \frac{\partial }{\partial u} J(y,{u}),{u}_\delta \right\rangle _{{\mathbb {R}}^m} + \left\langle \frac{\partial }{\partial u} \langle {p, e(y,{u})}\rangle _Z , {u}_\delta \right\rangle _{{\mathbb {R}}^m} \nonumber \\&= \langle {N \alpha ,{u}_\delta }\rangle _{{\mathbb {R}}^m} - \left\langle \int _0^T p(t)^\top \frac{\partial }{\partial u} g(t,{u},y(t)) \; dt, {u}_\delta \right\rangle _{{\mathbb {R}}^m} \end{aligned}$$

with $N= \text{ diag }(n_1, \ldots , n_m)$, and analogously for arbitrary $y_{\delta }\in Y$ we haveFrom the second KKT condition, we can derive the adjoint equation for $t \in \, [0,T]$ almost everywhere:11Using adjoint techniques and (6), it can be shown [21, Ch. 1.6.4] that

12 $$\begin{aligned} \langle { \nabla \hat{J} ({u}), {u}_\delta }\rangle _{{\mathbb {R}}^m}= & {} \frac{\partial }{\partial u} L(y({u}),{u},p({u})) {u}_\delta \nonumber \\= & {} \langle {N \alpha ,u_{\delta }}\rangle _{{\mathbb {R}}^m} - \left\langle \int _{0}^T p(t)^\top \frac{\partial }{\partial u}\tilde{r}(t,u) \; dt, u_{\delta }\right\rangle _{{\mathbb {R}}^m} \end{aligned}$$

for all ${u}_\delta \in {\mathbb {R}}^m$, and therefore, the variational inequality (9) is the first KKT condition with the derivative computed in (12). Having a formula for $\nabla \hat{J}({u})$, we can now solve ($\hat{P}$) numerically using well-known algorithms from finite-dimensional optimization, e.g., quasi-Newton methods.

#### Remark 3.2

- In contrast with the general, infinite-dimensional setting discussed in [21] we construct a finite-dimensional OCP by exploiting the design of PKPD models, see (6), (7). Therefore, the evaluation of $\nabla \hat{J}({u})$ comes at very low computational cost.
- Second-order sufficient conditions for a minimum can be verified by approximating the reduced Hessian [23, Thm. 5.3.2] with central differences of the gradients and computing the eigenvalues.

### Open-Loop Problems and Convergence Properties

Assuming that all model parameters are known prior to the optimization, we can solve ($\hat{P}$) in a so-called open-loop process in which the controls are computed iteratively until a certain stopping criterion is satisfied. It should be noted that during the iteration process there is no option to update parameters or react to external perturbations. We will use a quasi-Newton method with Armijo step size control where the Hessian $\nabla ^2 \hat{J}( {u})$ is approximated by projected BFGS updates named after Broyden, Fletcher, Goldfarb and Shanno. For more details on the step size strategy, the algorithm, local and global convergence results and second-order sufficient conditions we refer the reader to [23, Ch. 5.5.3].

### Numerical Adaptions for Impulse Optimal Control Problems

In case of oral / SC or IV bolus administration, the solution $y=y(t)$ of the impulse state equations (2)–(4) is smooth for $t \in [0,T] \backslash \{ t_{1,1}, \ldots , t_{m,n_m} \}$ and has jumps at the dosing points $t_{1,1} , \ldots , t_{m,n_m}$ with jump heights (5). Optimality conditions for impulse OCPs can be found in [19, Thm. 2.1]. However, following the typical approach in PKPD modeling, i.e., stopping the time integration at each dosing time point in order to add the dose to the corresponding compartment before continuing with the integration, ensures that

- the jump conditions are satisfied, i.e., the state equation is solved correctly,
- the adjoint equation (11) coincides with the one presented in [19, Thm. 2.1],
- the variational inequality (9) on which the stopping criterion in the algorithm is based is the same as the one in [19, Thm. 2.1].

Consequently, the impulse OCP is solved.

### The Nonlinear Model Predictive Control Method

For long-term treatments over several years, patient parameters can change over time, e.g., for pediatric patients due to developmental changes. Then, the OCP $(\hat{P})$ cannot be solved as a single open-loop problem and it is inevitable to apply nonlinear model predictive control (NMPC) techniques. The idea is to optimize the control ${u}$ on a sequence of overlapping open-loop problems with short time horizon $I_i \subset [0,T]$ covering the time interval instead of solving one open-loop problem on the full time interval. This approach allows parameter adaptions between consecutive open-loop problems. The time intervals $I_i$ should be chosen in a way such that one dose gets applied (possibly multiple times), and then, the time horizon is shifted to start just before the first dosing time point of the next dosing group; therefore, it is sometimes also called moving horizon method.

Let the *i*-th time interval $I_i := [t_i^0, t_i^f]$ start at $t_i^0 := t_{i,1}^-$ right before the first dosing time point of the *i*-th dosing group and end at $t_i^f := t_{i + \ell ,1}^-$ for $\ell \in {\mathbb {N}}$ right before the $(i+\ell )$-th dosing group begins. Then for $t_{m+1,1} := T$ the full time interval [0, *T*] is covered with short prediction horizons $I_i$ for $i = 1,\dotsc , m - \ell + 1$ each of them containing $\ell$ dosing groups.

Suppose we are at time $t_{i}^0$ for $i \le m - \ell + 1$ and consider the horizon $I_i$. Previously, the optimal doses $u_1, \dotsc , u_{i-1}$ and the associated optimal state trajectory $y^{i-1}$ from the last iteration were computed. Therefore, $y^{i}_0 := y^{i-1}(t_{i}^0)$ is set as initial value for the state equation on $I_i$

$$\begin{aligned} (y^i)'(t)&= g(t,{u}^{(i)},y^i(t)), \quad \text{ for } t \in I_i \text{ almost } \text{ everywhere } \\ y^i(t_{i}^0)&= y^{i}_0 \end{aligned}$$

where the control ${u}^{(i)}$ denotes ${u}^{(i)} =(u_i,\dotsc ,u_{i+\ell -1}) \in U_{{\mathrm{ad}}}^{(i)}$ and

$$\begin{aligned} U_{{\mathrm{ad}}}^{(i)} := \{ {u}^{(i)} \in {\mathbb {R}}^{\ell } \, | \, 0 \le {u}^{(i)} \le u_{\mathrm{max}}^{(i)} \} \end{aligned}$$

with $u_{\mathrm{max}}^{(i)}=((u_{\mathrm{max}})_i,\dotsc ,(u_{\mathrm{max}})_{i+\ell -1})$ being the corresponding admissible set. On the prediction horizon $I_i$, the cost functionalis used. As before, the reduced cost functional

$$\begin{aligned} \hat{J}_i ({u}^{(i)}) := J_i(y^i({u}^{(i)}),{u}^{(i)}) \end{aligned}$$

with the unique solution to the state equation $y^i({u}^{(i)})$ is introduced. The open-loop problem 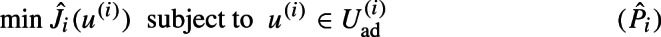


is solved as in Sect. 3.3 by computing an optimal control vector and predicting the dynamics on the time horizon $[t_{i}^0,t_{i}^f]$. Now, only the first component of the optimal control vector $u_i = ( u^{(i)})_1$ is applied and the time horizon is shifted to the next interval $I_{i+1}$ with initial condition $y^{i+1}_0:= y^i(t_{i+1}^0)$. Before solving $(\hat{P}_{i+1})$, it is possible to update parameters or react to unforeseen perturbations such as dosing errors or missed doses.

In Algorithm 1, a pseudocode for the described closed-loop technique is displayed.
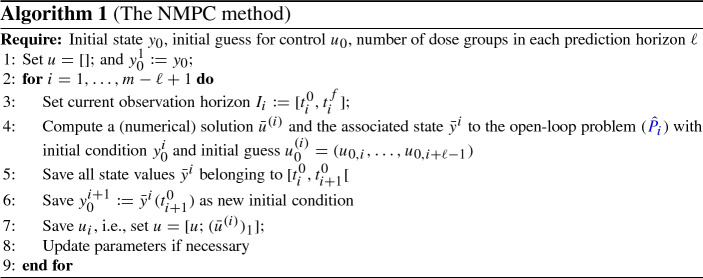


## Application of OptiDose to Relevant Examples of Pharmacokinetic–Pharmacodynamic Models and Presentation of Numerical Results

We present three relevant examples of PKPD models used in drug development and clinical pharmacology and apply the developed OptiDose algorithm to compute the optimal dosing regimen.

### The OptiDose Software

For the software OptiDose, the presented open- and closed-loop algorithms were implemented in MATLAB [24] using the built-in solver ode15s to solve the arising initial value problems. The open-loop problems were solved with the projected BFGS-Armijo method [23, Ch. 5.5.3]. All computations were performed on an ASUSTek computer with Intel(R) Core(TM) i7-7700HQ CPU processor with 2.80GHz and 16GB RAM.

**Fig. 1:**
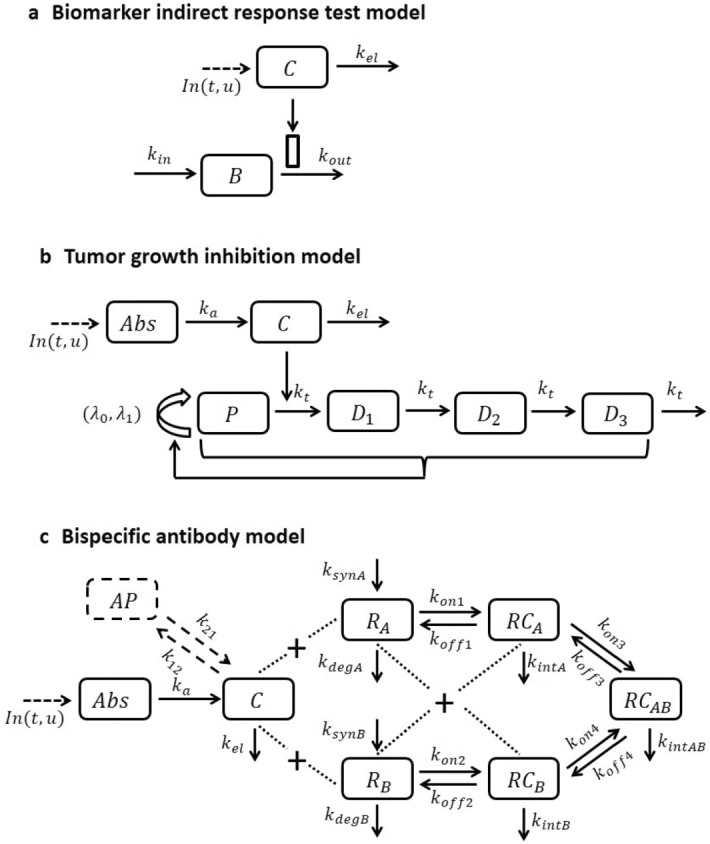
Model schematics for the three examples

### Biomarker Indirect Response Models: a Test Model for OptiDose

In Fig. 1 a, we display the model schematic for a test example where a biomarker *B* is elevated and a drug is administered to return those high biomarker levels to the normal range. We further assume that the disease cannot be cured, i.e., in absence of the drug the biomarker will return to its initial state $B^0$ at diagnosis. The test model is a so-called indirect response model (IDR) which is a fundamental tool in PKPD modeling, cf., e.g., [25, 26]. IDR models consist of a zero-order production rate $k_{\mathrm{in}}$ and a first-order elimination rate $k_{\mathrm{out}}$, and the drug stimulates or inhibits these rates usually with Michaelis–Menten type of terms [27]. Here the drug will be administered via IV bolus into the central compartment according to (4). The drug concentration *C* is described by a linear one-compartment model with drug elimination rate $k_{el}$. Maximal stimulating effect of the drug is $E_{\mathrm{max}}$, and the drug concentration to produce the half-maximal effect is $EC_{50}$. The PKPD model reads

$$\begin{aligned} \frac{d}{dt} C= & {} In(t,u) - k_{el} C , \qquad \qquad \qquad \qquad \quad \ \ \ \ C(0) = 0, \\ \frac{d}{dt} B= & {} k_{\mathrm{in}} - k_{\mathrm{out}} \left( 1 + \frac{E_{\mathrm{max}} C}{EC_{50} + C} \right) B, \, \qquad B(0) = B^0 = \frac{k_\mathrm{{in}}}{k_{\mathrm{out}}} . \end{aligned}$$

**Fig. 2:**
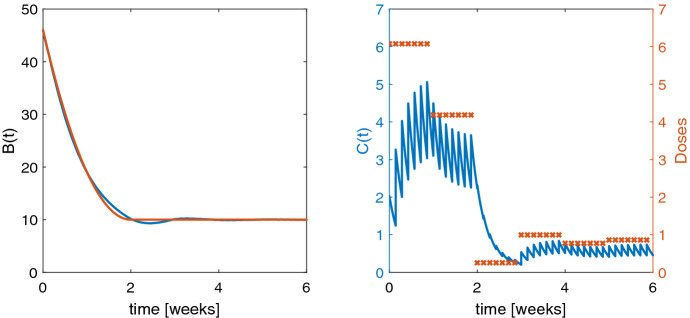
Left: pharmacodynamics of optimal solution in blue and reference function in red. Right: drug concentration for optimal dosing in blue and doses administered at dosing time points (red crosses)

A clinically realistic dosing scenario for non-hospitalized patients is that the doses are administered daily, but they change only every week ($n_i = 7$, $i=1, \ldots ,m$). The aim is to control the biomarker *B* to follow a predefined reference function characterized by

$$\begin{aligned} B_{\mathrm{ref}} (t) = {\left\{ \begin{array}{ll} \frac{1}{(7m_1)^2}\,(B^0 - B_{\mathrm{tar}})\, t^2 - \frac{2}{7m_1}\,(B^0 - B_{\mathrm{tar}})\, t + B^0, &{} t \le 7m_1 \\ B_{\mathrm{tar}}, &{} t > 7m_1 \end{array}\right. } \end{aligned}$$

providing a slow quadratic approach toward the target biomarker level $B_{\mathrm{tar}}$. We choose $\alpha = 0$ in the cost functional, a target value of $B_{\mathrm{tar}}=10$, a loading phase of $m_1 = 2$ weeks and $m=6$ weeks in total. Figure 2 shows the optimal solution on the left the actual and desired biomarker levels and on the right the optimal doses and the resulting drug concentration.

Starting from an initial guess of $u_0 = 1$ for all doses, the optimal open-loop solution $u^*$ was computed within 85 seconds and a cost functional value of $\hat{J}(u^*) = 3.89$ with norm of the gradient $5.9 \cdot 10^{-6}$ and all eigenvalues of the Hessian at the optimal solution are positive. The same optimal solution is found for different initial guesses on the doses.

However, in a real scenario the treatment will not stop after the above considered six weeks as we assumed the disease cannot be cured. The closed-loop algorithm with an observation horizon of, e.g., three weeks that gets shifted weekly reproduces the open-loop solution up to neglectable differences.

Individual parameter values used are $\theta _{ind} = (V, B^0, k_{\mathrm{out}}, k_{\mathrm{in}}, k_{el}, E_{\mathrm{max}},{EC}_{50}) =(3,46,0.02,0.92,0.49,8.8,0.81)$.

### Tumor Growth Inhibition Model

Proliferating tumor cells usually grow exponentially in the beginning and transition later to a linear growth, cf. [28, 29]. Depending on the tumor type, size and environment, a saturation of the growth can be observed; however, this is neglected in the following example. Many drugs act in a cytotoxic manner, meaning that the tumor cells are attacked by the drug. Attacked cells then undergo apoptosis until they eventually die. The presented example is based on preclinical oncology drug development in mice, see [29] for details and Fig. 1 b for the model schematic. The structure of the model is widely applied in industry and academia for these type of experiments.

Let *P* be the proliferating tumor cells with an exponential growth rate $\lambda _0$ and a linear growth rate $\lambda _1$ [29]. The drug *C*, orally administered into an absorption compartment *Abs* with absorption rate $k_a$, acts on the proliferating cells with a linear drug effect term with potency $k_\mathrm{pot}$. The apoptotic cell population is described with three transit compartments $D_1$, $D_2$, $D_3$ [30], each reflecting a certain age stage of the apoptotic cells with transit rate $k_t$. The sum of the proliferating and apoptotic cells is the total tumor weight $W=P+D_1+D_2+D_3$. The PKPD model reads

$$\begin{aligned} \frac{d}{dt} Abs= & {} In(t,u) - k_{{a}}Abs , \quad Abs(0)=0, \\ \frac{d}{dt} C= & {} k_{{a}}\frac{Abs}{V} - k_{{el}}C, \qquad C(0)=0, \\ \frac{d}{dt} P= & {} \frac{2\lambda _0 \lambda _1 P^2}{(\lambda _1 + 2 \lambda _0 P)W} - k_{\mathrm{pot}}C \cdot P, \qquad \, P (0) = P^0, \\ \frac{d}{dt} D_1= & {} k_{\mathrm{pot}}C \cdot P - k_{t}D_1, \quad D_1 (0) = 0, \\ \frac{d}{dt} D_i= & {} k_{t}( D_{i-1} - D_i ), \qquad D_i (0) = 0, \; i=2,3. \end{aligned}$$

First, the tumor is grown to a specific size, then the drug is administered daily ($n_i =1$ for all *i*) from day 12 to 28. The aim is to decrease the tumor weight *W* toward zero. For the reference function, we choose a sigmoid shape starting at 0.5 on day 12 and tending to zero:

$$\begin{aligned} W_\mathrm{ref} (t)= \frac{0.25 \,(\exp (2) - \exp (-2)) }{0.5 \,(\exp (2) - 3\exp (-2)) + \exp (0.5t - 8)}, \quad t \ge 12 \end{aligned}$$

As the drug is acting on the proliferating tumor cells via $k_{\mathrm{pot}}C \cdot P$ in the third equation in the PKPD model, its impact decreases as the tumor size shrinks toward 0. In fact, the problem loses its controllability meaning significantly different doses, e.g., 10, 100, 1000, achieve nearly the same pharmacodynamic behavior. Naturally, small doses are preferred which is why we choose $\alpha = 10^{-7} > 0$ in the cost functional in favor of lower doses.

Moreover, we observe that large changes in the doses result in only very small changes in the cost functional as well as in the gradient which might result in stopping criteria being satisfied already despite the minimum was not reached yet. Therefore, for numerical reasons, we minimize the theoretically equivalent cost functional

$$\begin{aligned} \hat{J}_\gamma (u) = \gamma \cdot \hat{J}(u) \end{aligned}$$

by including a scaling factor $\gamma > 0$ which pulls through to the gradient and hereby tightens the stopping criterion that the norm of the projected gradient is small enough. For the presented example, we choose a scaling factor of $\gamma = 100$.

**Fig. 3:**
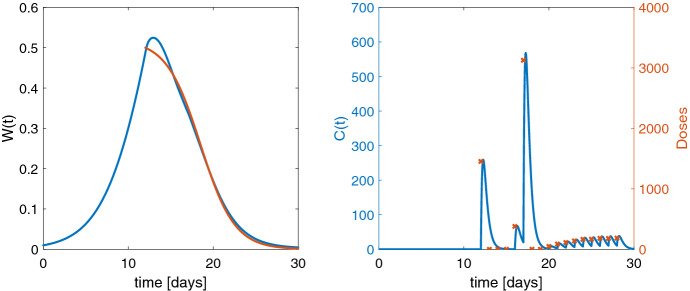
Left: optimal closed-loop solution for an observation horizon of 7 days in blue and the sigmoid reference function in red. Right: drug concentration for optimal dosing in blue (computed for an observation horizon of 7 days) and administered drug doses $\hat{u}$ (red crosses)

The initial guess for each dose is ${u}_0 = 0$. We solve the OCP with the NMPC (i.e., closed-loop) method as described in Algorithm 1 with an observation horizon of $\ell = 7$ doses which is shifted daily after applying the first of the computed optimal doses. Altogether, we find the closed-loop solution $\hat{u}$, see Fig. 3 within 175 seconds with cost functional value $\hat{J}_\gamma (\hat{{u}}) = 0.2165$ and its norm of the gradient $6.52 \cdot 10^{-5}$. In the present case without model parameter changes the closed-loop solution $\hat{u}$ is a suboptimal solution compared to the open-loop solution $u^*$, i.e., computing the optimal dosing regimen for days 12 to 28 at once.

**Fig. 4:**
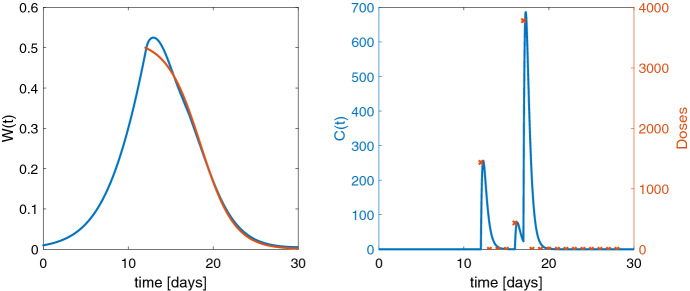
Left: optimal solution from open loop in blue and the sigmoid reference function in red. Right: drug concentration for optimal dosing in blue (computed with open loop) and administered drug doses $u^*$ (red crosses)

However, the open loop yields only slightly better results. Within 250 seconds we get $\hat{J}_\gamma ( {u}^*) = 0.2028$ with norm of the projected gradient $7.66 \cdot 10^{-10}$ and all eigenvalues of the reduced Hessian are positive. The open-loop solution (see Fig. 4) provides higher doses on days 16 and especially 17 whose necessity the shorter horizon does not see, but the closed loop (Fig. 3) compensates this with larger doses in the following days. The difference in the cost functional values is small.

Numerical computations with a variety of different initial values confirm that $u^*$ is the unique optimal control. In addition, the algorithm shows good non-local convergence properties. Both findings fit in the framework of optimal control problems with convex cost functionals.

The parameter values $\theta =(V,k_{{a}}, k_{{el}}, \lambda _0 , \lambda _1, k_{t}, k_{\mathrm{pot}}, P^0)$ taken from [29] are $V=2.79, k_{{a}}=5, k_{{el}}= 2.53, \lambda _0 = 0.194, \lambda _1 = 0.246, k_{t}= 0.666, k_{\mathrm{pot}}= 0.0077$ and the initial state $P^0 = 0.0098$.

### Binding Kinetics of a Bispecific Antibody and Formation of the Drug–Receptor Complex

Bispecific antibodies (BsAbs) are promising drug candidates in immuno-oncology [31], and currently extensive research is performed by both academia and pharmaceutical companies. A BsAb is an artificial protein and exerts the effect, e.g., by bridging effector T cells and cancer cells. The idea of BsAbs is to use the own human immune system to attack tumor cells instead of administering cytotoxic drugs as in the previous example.

Mechanistic modeling of a BsAb is a sophisticated task and includes characterization of many different binding kinetics [32, 33]. A BsAb *C* binds to two different receptors $R_A$ and $R_B$ forming the two binary drug–receptor complexes $RC_A$ and $RC_B$. Both of these complexes further cross-bind to the other receptors to form the ternary complex $RC_{AB}$ which finally drives the drug effect. The absorption process of the BsAb is described by *Abs*. The full BsAb model is schematically displayed in Fig. 1 c and reads (see [13] for details):

$$\begin{aligned} \frac{{d}}{dt}C&= k_{{\mathrm{off}}1}RC_{A}+ k_{{\mathrm{off}}2}RC_{B}- (k_{{el}}+ k_{\mathrm{on}1}R_{A}+ k_{\mathrm{on}2}R_{B}+ k_{12})C \\&\quad + k_{21}\frac{AP}{V} + k_{{a}}\frac{Abs}{V}, \\ \frac{{d}}{dt}R_{A}&= k_{\mathrm{syn}A}- (k_{\mathrm{deg}A}+k_{\mathrm{on}1}C+k_{\mathrm{on}4}RC_{B}) R_{A}+ k_{{\mathrm{off}}1}RC_{A}+ k_{{\mathrm{off}}4}RC_{AB}, \\ \frac{{d}}{dt}R_{B}&= k_{\mathrm{syn}B}- (k_{\mathrm{deg}B}+k_{\mathrm{on}2}C+ k_{\mathrm{on}3}RC_{A}) R_{B}+ k_{{\mathrm{off}}2}RC_{B}+ k_{{\mathrm{off}}3}RC_{AB}, \\ \frac{{d}}{dt}AP&= k_{12}C\cdot V - k_{21}AP, \\ \frac{{d}}{dt}Abs&= -k_{{a}}Abs+ In (t,u) \end{aligned}$$

and the equations for the complexes

$$\begin{aligned} \frac{{d}}{dt}RC_{A}&= k_{\mathrm{on}1}C\cdot R_{A}- (k_{{\mathrm{off}}1}+k_{\mathrm{int}A}) RC_{A}- k_{\mathrm{on}3}R_{B}\cdot RC_{A}+ k_{{\mathrm{off}}3}RC_{AB}, \\ \frac{{d}}{dt}RC_{B}&= k_{\mathrm{on}2}C\cdot R_{B}- (k_{{\mathrm{off}}2}+k_{\mathrm{int}B}) RC_{B}- k_{\mathrm{on}4}R_{A}\cdot RC_{B}+ k_{{\mathrm{off}}4}RC_{AB}, \\ \frac{{d}}{dt}RC_{AB}&= k_{\mathrm{on}4}R_{A}\cdot RC_{B}+ k_{\mathrm{on}3}R_{B}\cdot RC_{A}- (k_{{\mathrm{off}}3}+k_{{\mathrm{off}}4}+k_{\mathrm{int}AB}) RC_{AB}. \end{aligned}$$

Briefly, the $k_\mathrm{on}$ and $k_{\mathrm{off}}$ are binding rates, $k_\mathrm{syn}$ and $k_\mathrm{deg}$ production, resp. degradation rates, $k_\mathrm{int}$ internalization rates, $k_\mathrm{el}$ is the elimination rate of the BsAb and $k_{12}$, $k_{21}$ describe the transfer to a peripheral compartment $AP$. Antibody drugs have a very long half-life and are often administered via SC injection, cf. (4). Therefore, we consider the time interval [0, 140] days with administration of the same dose into the absorption compartment $Abs$ at days 0, 48, 96, i.e., $n_1 =3$. Initially, we assume the system to be at baseline, i.e., $R_{A}(0) = k_{\mathrm{syn}A}/ k_{\mathrm{deg}A}$ and $R_{B}(0) = k_{\mathrm{syn}B}/ k_{\mathrm{deg}B}$ and all others are 0. Throughout the whole interval, our goal is to keep the ternary complex $RC_{AB}$ at the best possible reference value $\min (k_{\mathrm{syn}A}/ k_{\mathrm{deg}A}, \, k_{\mathrm{syn}B}/ k_{\mathrm{deg}B})$. In the cost functional, we choose $\alpha = 0$.

**Fig. 5:**
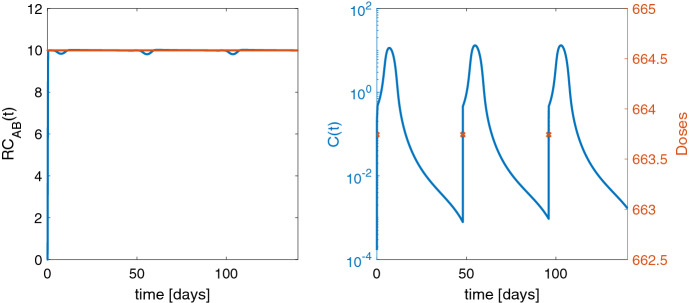
Left: ternary complex $RC_{AB}$ for the optimal solution in blue and reference value 10 in red. Right: concentration level $C$ as semilogarithmic plot for the optimal solution in blue and administered dose $u^*$ at dosing time points (red crosses)

Starting from an initial guess for the dose of 800, we find the optimal (open-loop) solution ${u}^* = 663.78$ within 6 iterations of BFGS-Armijo method and 21 seconds. The optimal doses and resulting ternary complex are shown in Fig. 5. The cost functional value at the optimal solution is $\hat{J} ({u}^*) = 5.0$, the norm of its gradient is $1.2 \cdot 10^{-8}$, and its Hessian is positive. Starting from an initial guess far from the optimal solution resulting in a large cost functional value, e.g., $u_0 = 0$ with $\hat{J}(u_0) = 7000$ yields the same optimal solution within 22 iterations and 32 seconds, i.e., the algorithm shows numerical robustness.

The parameter values taken from a simulation study [13] are $k_{{el}}= 0.1, k_{\mathrm{on}1}= 10, k_{{\mathrm{off}}1}= 0.01,$ $k_{\mathrm{on}2}= 1, k_{{\mathrm{off}}2}= 0.01, k_{\mathrm{on}3}= 1, k_{{\mathrm{off}}3}= 0.01, k_{\mathrm{on}4}=10,$ $k_{{\mathrm{off}}4}= 0.01, k_{\mathrm{syn}A}= 1, k_{\mathrm{deg}A}= 0.1, k_{\mathrm{syn}B}= 10, k_{\mathrm{deg}B}=0.1,$ $k_{\mathrm{int}A}= 0.05, k_{\mathrm{int}B}= 0.05, k_{\mathrm{int}AB}= 0.1, k_{12}= 0, k_{21}= 0.03, k_{{a}}= 0.2$ and $V = 3$.

## Conclusions

We have set up an OCP which is especially designed for PKPD models using the doses as finite-dimensional control variables (instead of optimizing the drug concentration). Therefore, the reduced OCP can be solved by robust algorithms from finite-dimensional optimization such as quasi-Newton methods. An efficient calculation of the required derivatives is ensured by the use of adjoint techniques. In addition, we incorporated closed-loop (NMPC) algorithms which provide, in particular in case of long-term treatments, the potential to guide patients safely along a desired reference function $h_\mathrm{ref}$. Application to a variety of PKPD models shows the robustness and efficiency of the software. Thus, the presented OptiDose code is a widely usable tool for computing the individualized optimal drug dosing regimen in PKPD and, to our knowledge, the first calculating the doses directly.

On the other hand, we still need to address some PKPD issues, e.g., a rigorous sensitivity analysis of the computed optimal doses with respect to uncertainty in the estimation of the parameter values. Suitable measures to identify sensitive parameters should be discussed.

Another important issue will be how to handle negative side effects from drug treatment such as myelosuppression in oncology: The administration of cytotoxic drugs to attack a tumor also suppresses the production of new blood cells in the bone marrow leading to low levels of circulating blood cells. As white blood cells play an important role in the immune system, they need to stay above a certain threshold in order to not risk the life of the patient. Mathematically, this means to include so-called state constraints in the OCP making it usually much harder to solve numerically. A promising way is to apply augmented Lagrangian techniques using an additional penalization term in the cost functional.

Furthermore, in daily clinical routine the available oral doses are typically restricted to certain sizes which leads to discrete ODE-constrained OCPs. The presented open issues are both mathematically challenging and of huge interest in applications and provide a wide field for future research.

## Data Availability

Data sharing is not applicable to this article as no datasets were generated or analyzed during the current study.
